# De-acetylation and degradation of HSPA5 is critical for E1A metastasis suppression in breast cancer cells

**DOI:** 10.18632/oncotarget.2510

**Published:** 2014-09-25

**Authors:** Yi-Wen Chang, Hsin-An Chen, Chi-Feng Tseng, Chih-Chen Hong, Jui-Ti Ma, Mien-Chie Hung, Chih-Hsiung Wu, Ming-Te Huang, Jen-Liang Su

**Affiliations:** ^1^ National Institute of Cancer Research, National Health Research Institutes, Zhunan, Miaoli Country, Taiwan; ^2^ Graduate Institute of Clinical Medicine, College of Medicine, Taipei Medical University, Taipei, Taiwan; ^3^ Division of General Surgery, Department of Surgery, Shuang Ho Hospital, Taipei Medical University, New Taipei City, Taiwan; ^4^ Graduate Program of Biotechnology in Medicine College of Life Science, National Tsing Hua University, Hsinchu, Taiwan; ^5^ Graduate Institute of Cancer Biology, China Medical University, Taichung, Taiwan; ^6^ Center for Molecular Medicine, China Medical University Hospital, Taichung, Taiwan; ^7^ Department of Biotechnology, Asia University, Taichung, Taiwan; ^8^ Department of Molecular and Cellular Oncology, The University of Texas MD Anderson Cancer Center, Houston, Texas, USA

**Keywords:** acetylation, adenovirus type 5 E1A, HSPA5/GRP78/Bip, metastasis, ubiquitination

## Abstract

Elevated expression of heat shock protein 5 (HSPA5) promotes drug resistance and metastasis and is a marker of poor prognosis in breast cancer patients. Adenovirus type 5 E1A gene therapy has demonstrated antitumor efficacy but the mechanisms of metastasis-inhibition are unclear. Here, we report that E1A interacts with p300 histone acetyltransferase (HAT) and blocks p300-mediated HSPA5 acetylation at K353, which in turn promotes HSPA5 ubiquitination by GP78 (E3 ubiquitin ligase) and subsequent proteasome-mediated degradation. Our findings point out the Ying-Yang regulation of two different post-translational modifications (ubiquitination and acetylation) of HSPA5 in tumor metastasis.

## INTRODUCTION

Breast cancer is the most commonly diagnosed cancer and the leading cause of cancer-related deaths in women worldwide [1]. Current therapeutic options for treating breast cancer have focused on various combinations of surgery, chemotherapy, targeted therapy and radiation treatment [2]. Despite improvements, metastatic breast cancer responds poorly to conventional therapy. Therefore, further investigation of the molecular mechanisms and identification of specific targets in the metastatic process are critical for developing more effective breast cancer therapies.

Recent studies have indicated that the activation of the unfolded protein response (UPR) is essential in solid tumors and correlates with aggressive cancer types [3]. As protein synthesis increases in fast-growing solid tumors, cancer cells also require increased endoplasmic reticulum (ER) capacity and function [4]. Heat shock protein 5 (HSPA5), also known as GRP78/BiP, is a major ER chaperone that responds to UPR and is involved in many cellular processes that promote proper protein folding and prevent aggregation of newly synthesized proteins [5]. HSPA5 overexpression has been reported in many tumor types, including lung [6], breast [7, 8], prostate [9], colon [10], stomach [11] and liver [12]. HSPA5 exhibits oncogenic activities by promoting tumor proliferation, survival, metastasis, and drug resistance and is associated with malignancy and poor prognosis [13, 14]. Mice with HSPA5 haploinsufficiency have decreased mammary tumor growth, development, and metastasis as well as increased survival [13, 15]. HSPA5 knockdown also inhibits tumor cell metastasis *in vitro* and *in vivo* [11, 14].

Adenovirus type 5 E1A possesses anti-cancer activity and has been tested in multiple clinical trials, including trials for breast, ovarian and head and neck cancers [16-19]. E1A exerts its anti-cancer activity through a diverse range of mechanisms, such as increasing drug sensitivity and decreasing metastasis through the down-regulation of HER2/neu [20, 21], eliciting the production of apoptotic molecules through p38 activation [22], sensitizing cells to radiation-induced apoptosis through IKK activity suppression and IκB degradation [23], decreasing miR-520h levels and the EMT marker TWIST to inhibit metastasis [24], promoting the transformation of malignant cancer cells into a benign epithelial phenotype and inducing anoikis-sensitization through interactions with its co-repressor CtBP to activate E-cadherin and repress ZEB expression [25], and increasing chemo-sensitization by stabilizing FOXO3a through ubiquitin-proteolysis pathway inhibition [26].

Based on the function of HSPA5 in cancer progression, HSPA5 may represent a therapeutic cancer therapy target. We sought to examine any potential connections between HSPA5 and E1A-mediated anti-cancer activities. In this study, we demonstrate that the E1A/p300 interaction inhibits HSPA5 acetylation at K353 and promotes GP78 the E3 ligase-mediated ubiquitination of HSPA5 and its subsequent degradation, which leads to decreased breast cancer cell metastasis *in vitro* and *in vivo*.

## RESULTS

### HSPA5 is critical for E1A-mediated suppression of cell mobility

**Figure 1:**
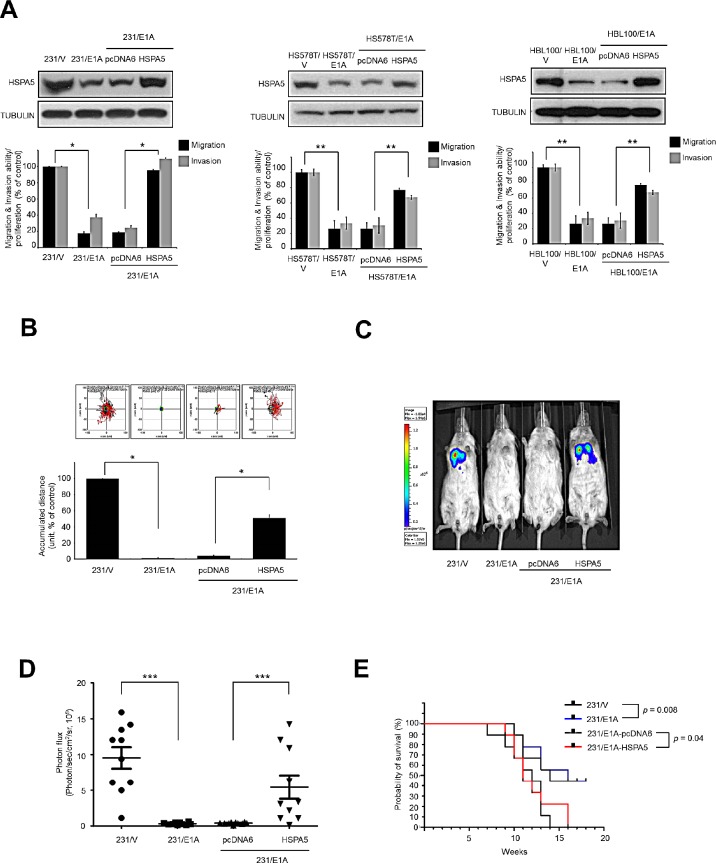
HSPA5 repression is required for E1A-mediated metastasis suppression (A) Three breast cancer cell lines were stably transfected with control vector (231/V, HS578T/V, HBL100/V) or E1A-expressing vector (231/E1A, HS578T/E1A, HBL100/E1A) with pcDNA6 or HSPA5 constructs. The expression of HSPA5 was analyzed by Western blot assay; cell migration and invasive ability were measured by transwell migration and matrigel invasion assay. Percentage of cell migration and invasion/proliferation is shown relative to control vector group. Data shown are mean ± s.e.m. of three independent experiments performed in triplicate. *p < 0.05; **p < 0.01 by *t*-test. (B) Measurement of migration ability by a time-lapse cell tracker migration assay. Top, representative images from the time-lapse cell tracker migration assay. Bottom, quantification of the time-lapse cell tracker migration assay results. Data shown are mean ± s.e.m. of three independent experiments performed in triplicate. *p < 0.05 by *t*-test. (C) 231/V, 231/E1A, 231/E1A/pcDNA6 and 231/E1A/HSPA5 cells stably expressing a luciferase reporter vector were injected into immunodeficient SCID mice through tail vein injection. Bioluminescence imaging of lung colonization measured 8 weeks after transplantation. (D) Values of photon flux from SCID mice were implanted 231/V, 231/E1A, 231/E1A/pcDNA6 and 231/E1A/HSPA5 cells stably expressing a luciferase reporter vector through tail vein injection; each data point represents mean ± s.e.m. (*n* = 10/group). ***p < 0.001 by *t*-test. (E) Kaplan-Meier plots of overall survival in SCID mice injected with 231/V, 231/E1A, 231/E1A/pcDNA6, or 231/E1A/HSPA5 cells (*n* = 10/group, log-rank test).

Based on the function of HSPA5 as a molecular target in cancer metastasis and progression, we sought to investigate whether HSPA5 was a target of E1A in breast cancer cells. We transfected E1A or control expression vectors into a panel of breast cancer cell lines to investigate the effects of E1A on HSPA5 expression and found that E1A suppressed HSPA5 expression and cell migration/invasion in three breast cancer cell lines (MDA-MB-231, HS578T and HBL100 cells) (Fig. 1A). In addition, we used an HSPA5-overexpressing or control vector ectopically expressed in MDA-MB-231 cells stably expressing E1A (231/E1A) to investigate the effects of HSPA5 on the E1A-mediated suppression of cancer cell migration/invasion (Fig. 1A). Consistent with the results in the 231/E1A cell system, HSPA5 overexpression in two other E1A-expressing cell lines (HS578T/E1A and HBL100/E1A) restored cell migration and invasion. Results from transwell and time-lapse cell tracker migration assays also indicated that HSPA5 overexpression attenuated the E1A-mediated suppression of cell mobility (Fig. 1B). Next, we used the established stable transfectants to further investigate the effects of HSPA5 on E1A-mediated anti-tumor activity in a xenograft tumor model. This model used mice that had been administered HSPA5-transfected E1A stable clones by tail-vein injection and utilized a bioluminescence system to detect metastasis development in mice. Compared with 231/vector control-bearing mice, lung colony formation was markedly reduced in 231/E1A-bearing mice but not in 231/E1A/HSPA5-bearing mice (Fig. 1C and 1D). These observations suggest that HSPA5 is critical for migration/invasion, tumorigenesis, and lung colonization of breast cancer cells and that E1A-suppressed HSPA5 is required for metastasis inhibition. In these groups, 231/E1A-bearing mice exhibited increased overall survival compared with 231/V-bearing mice; however, the increase in overall survival was significantly diminished in 231/E1A/HSPA5-bearing mice compared with 231/E1A-bearing mice (Fig. 1E). These data suggest that HSPA5 is critical for the E1A-mediated suppression of breast cancer cell mobility.

### E1A induces the ubiquitination and proteasomal degradation of HSPA5

**Figure 2:**
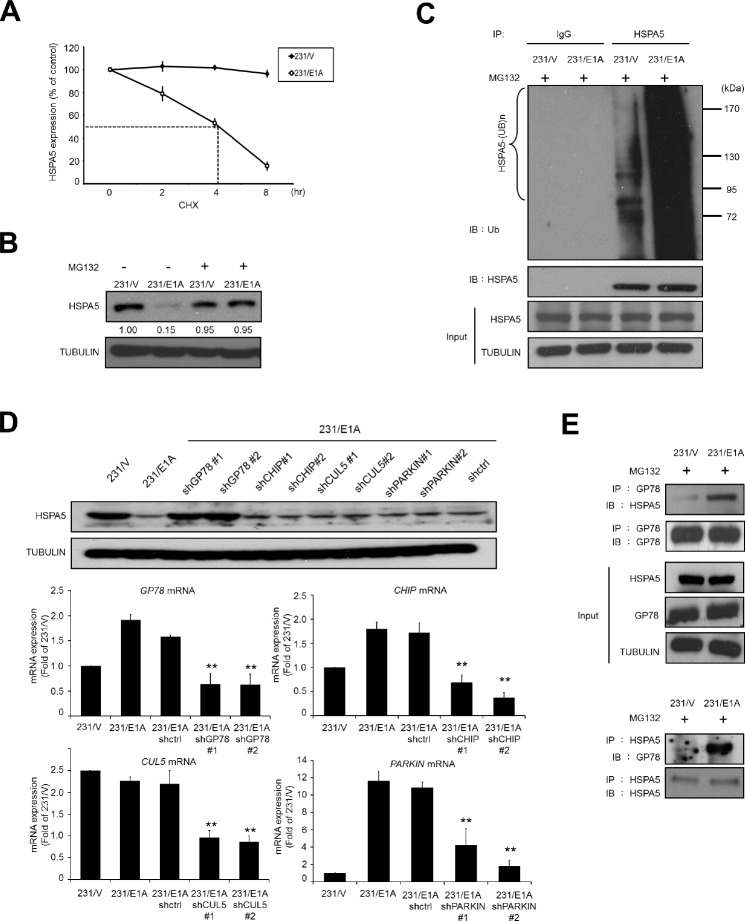
E1A decreases HSPA5 expression through GP78-mediated proteasomal degradation (A) Determination of the protein stability of HSPA5 in 231/V and 231/E1A cells. 231/V and 231/E1A cells were treated with 100 μg/ml cycloheximide (CHX) for the indicated times, and then following by Western blot analysis. Quantification of HSPA5 expression was performed three independent experiments using the Image J system and was normalized to the vehicle control. (B) 231/V and 231/E1A cells were treated with or without the proteasome inhibitor MG132 (5 μM) for 12 h, and HSPA5 expression was analyzed by Western blot analysis. The fold change in the protein expression is shown below the lanes, with the expression levels normalized to lane 1. (C) 231/V and 231/E1A cells were treated with the proteasome inhibitor MG132 (5 μM) for 12 h. Total cell lysates were prepared for *in vivo* ubiquitination assay. IgG was used as a control for the immunoprecipitation (IP) analysis. (D) 231/E1A cells were transfected with the indicated shRNAs, and HSPA5 expression was measured by Western blot analysis (top) and real-time RT-PCR (bottom). Data shown are mean ± s.e.m. of three independent experiments performed in triplicate. **p < 0.01 by *t*-test. (E) 231/V and 231/E1A cells were treated with the proteasome inhibitor MG132 (5 μM) for 12 h, and total cell lysates were harvested for IP and Western blot analysis.

Because E1A suppressed cell migration and invasion through regulation of HSPA5 expression (Fig. 1A), we further examined whether E1A regulated HSPA5 stability. Cells (231/V and 231/E1A) were treated with the protein synthesis inhibitor cycloheximide (CHX), and HSPA5 expression was measured by Western blot analysis. As shown in Fig. 2A, E1A expression markedly reduced the half-life of HSPA5 (< 4 h). We then analyzed endogenous HSPA5 protein expression in the presence of the 26S proteasome inhibitor MG132 to determine how HSPA5 was degraded in response to E1A. We found that the E1A-mediated downregulation of HSPA5 was restored by MG132 treatment (Fig. 2B), suggesting that E1A mediates HSPA5 degradation via the ubiquitin proteasome system. Additionally, immunoprecipitation (IP) using an anti-HSPA5 antibody followed by an anti-ubiquitin antibody revealed that E1A expression increased HSPA5 ubiquitination in breast cancer cells (Fig. 2C). Next, we sought to determine which E3 ubiquitin ligase was involved in the HSPA5 degradation process. Because of the oncogenic properties and ER localization of HSPA5, we screened a series of ER-associated degradation (ERAD)-related E3 ubiquitin ligases, including GP78, CHIP, CUL5, and PARKIN [27-30]. A specific shRNA knockdown of GP78 but not CHIP, CUL5, or PARKIN restored HSPA5 expression in 231/E1A cells (Fig. 2D). To gain further mechanistic insight into how GP78 regulates HSPA5 ubiquitination, we analyzed their interaction by co-immunoprecipitation. We found that in the presence of MG132, E1A enhanced the physical interaction between GP78 and HSPA5 (Fig. 2E). These results suggest that GP78 is a novel E3 ubiquitin ligase involved in E1A-mediated HSPA5 regulation through its binding to HSPA5.

### GP78 is involved in the E1A-mediated degradation of HSPA5 and metastasis inhibition

**Figure 3:**
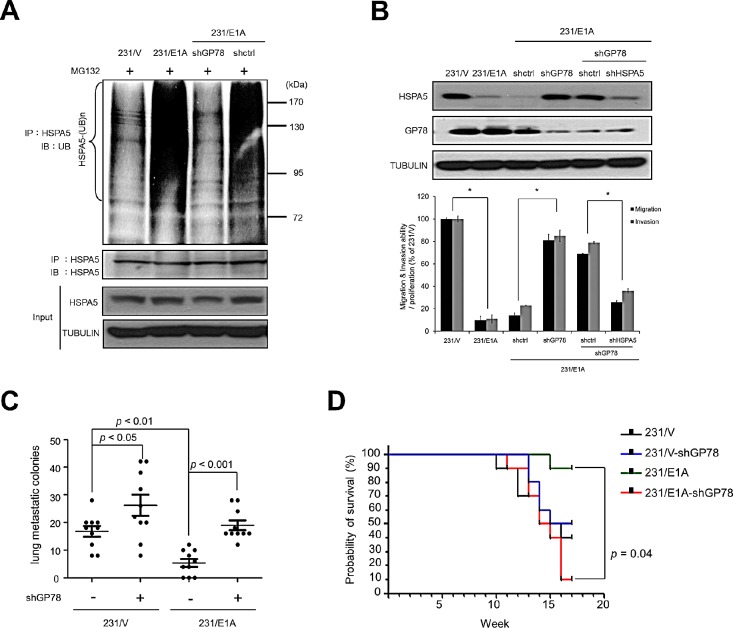
GP78 is an E3 ubiquitin ligase of HSPA5 and a tumor suppressor of breast cancer (A) 231/E1A cells were transfected with shGP78 (231/E1A/shGP78) and shcontrol (231/E1A/shctrl) and submitted to an *in vivo* ubiquitination assay. (B) The expression of HSPA5 and GP78 in 231/E1A/shctrl cells, 231/E1A/shGP78 cells and 231/E1A/shGP78 cells transfected with shctrl or shHSPA5 were analyzed by Western blot analysis (top). Transwell migration assays and Matrigel invasion assays were also performed on these cells (bottom). Percentage of cell migration and invasion/proliferation is shown relative to control vector. Data shown are mean ± s.e.m. of three independent experiments performed in triplicate. *p < 0.05 by *t*-test. (C) 231/V, 231/V/shGP78, 231/E1A and 231/E1A/shGP78 cells stably expressing a luciferase reporter gene were i.v. injected into immunodeficient SCID mice. Lung metastatic colonies were counted with a stereoscopic microscope (*n* = 10/group, *t*-test). (D) Kaplan-Meier plots of overall survival in experimental SCID mice injected with 231/V, 231/V/shGP78, 231/E1A, or 231/E1A/shGP78 cells (*n* = 9/group, log-rank test).

To examine whether GP78 was involved in *in vivo* E1A-mediated HSPA5 ubiquitination, endogenous GP78 was knocked down with shRNA. HSPA5 ubiquitination decreased in 231/E1A cells with a GP78 knockdown compared with control shRNA (Fig. 3A, lines 3 and 4). Because HSPA5 expression is critical for cancer cell mobility, we also examined whether GP78 affected cell migration and invasion. GP78 knockdown restored HSPA5 expression and also increased 231/E1A cell migration and invasion (Fig. 3B, lines 4 and 3). However, the enhanced cell migration and invasion was reduced by knocking down HSPA5 in GP78-silenced 231/E1A cells (Fig. 3B, lines 6 and 5), which suggests that GP78 is required for the E1A-mediated suppression of cell migration and invasion through HSPA5 proteolysis.

To further determine the effects of GP78 on E1A-mediated anti-metastasis activity in a xenograft tumor model, mice received stably transfected cells (231/V, 231/V/shGP78, 231/E1A, and 231/E1A/shGP78) by tail-vein injection. Seven out of ten mice injected with 231/E1A cells exhibited metastasis, but a GP78 knockdown in 231/E1A cells enhanced metastasis in all 10 mice. Mice bearing 231/E1A-shGP78 xenografts had a significantly increased number of metastatic nodules in the lungs compared with the control (Fig. 3C ). The survival rate of mice bearing 231/E1A-shGP78 xenografts was also significantly diminished compared with the control (*p =* 0.04; Fig. 3D ). These results suggest that GP78 E3 ubiquitin ligase activity is required for HSPA5 ubiquitination and involved in the regulation of E1A-mediated metastasis inhibition.

### E1A binds to p300 to prevent p300-induced HSPA5 acetylation and facilitates HSPA5 ubiquitination

**Figure 4:**
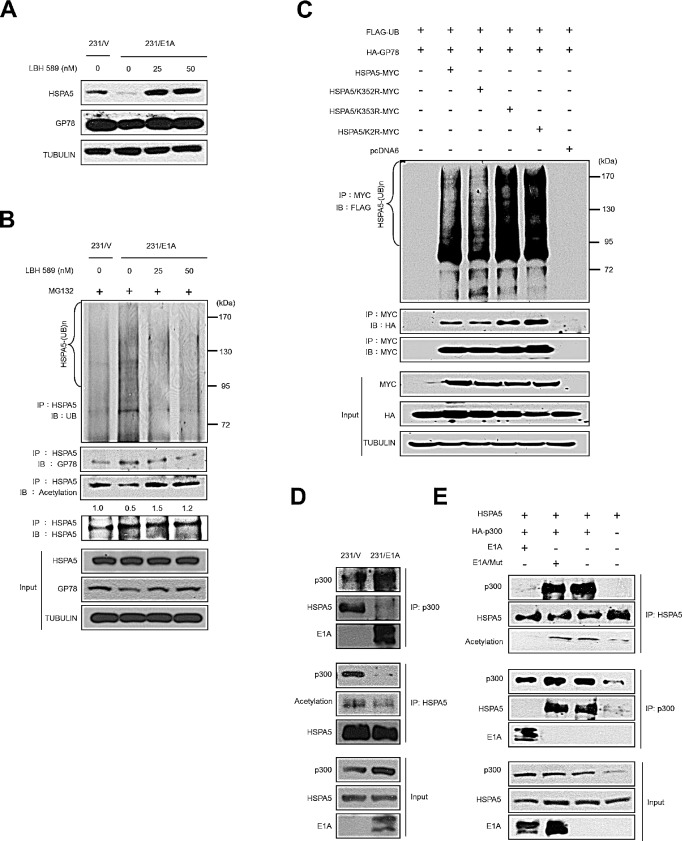
E1A prevents p300-mediated HSPA5 acetylation and promotes ubiquitin-dependent proteolysis of HSPA5 (A) The expression levels of HSPA5 and GP78 in 231/V and 231/E1A cells exposed to the indicated dosages of an HDAC inhibitor (LBH589) were detected by Western blot analysis. (B) *in vivo* ubiquitination assay in 231/V and 231/E1A cells treated with LBH589. (C) HeLa cells were transfected with the indicated constructs, and an *in vivo* ubiquitination assay was performed. (D) 231/V and 231/E1A cells were treated with MG132 (5 μM), followed by IP and Western blot analysis. (E) HeLa cells were transfected with HSPA5 or HA-p300 and either E1A or E1A/Mut (loss of binding ability to p300), cell lysates were harvested for IP and Western blot analysis.

Recent studies have suggested that HSPA5 acetylation is associated with the binding of HSPA5 and ER stress mediators that prolongs UPR [31]. However, whether acetylated HSPA5 exhibits changes in protein stability is unclear. To address this question, 231/V and 231/E1A cells were treated with a histone deacetylase inhibitor (HDACi), LBH589, and HSPA5 and GP78 expression was examined by Western blot analysis. In 231/E1A cells, treatment with LBH589 increased HSPA5 expression but not GP78 expression (Fig. 4A). We further examined whether HSPA5 acetylation affected its ubiquitination. Thus, 231/V and 231/E1A cells were exposed to LBH589, and HSPA5 ubiquitination was analyzed by IP and Western blot analysis. As shown in Fig. 4B, 231/E1A cells exhibited increased HSPA5 ubiquitination and reduced HSPA5 acetylation compared with 231/V cells. More importantly, LBH589 treatment increased HSPA5 acetylation and decreased the binding of the E3 ubiquitin ligase GP78 to HSPA5 and subsequent HSPA5 ubiquitination (Fig. 4B).

Eleven putative acetylated lysine residues in HSPA5 have previously been identified by mass spectrometry-based proteomic analyses [31]. However, whether the acetylated lysine residues in HSPA5 interfered with HSPA5 ubiquitination remained uncertain. To further confirm whether the acetylated lysine residues of HSPA5 reduce ubiquitylated HSPA5, various HSPA5 constructs were transfected into HeLa cells, and their ubiquitination levels were monitored by IP and Western blot analysis. HSPA5 ubiquitination in wild-type HSPA5-transfected cells increased compared with untransfected cells (Fig. 4C, lines 2 and 1). The ubiquitination levels of the K352R mutant, which cannot be acetylated, were similar to the wild-type HSPA5 levels (Fig. 4C, lines 2 and 3). In contrast, ubiquitination of the K353R mutant was increased compared with wild-type HSPA5 levels, and its association with GP78 was also enhanced (Fig. 4C, line 4). Moreover, transfection of the K352R/353R double mutant (HSPA5/K2R) also increased ubiquitination of HSPA5 and its interaction with GP78 (Fig. 4C, line 5). These results imply that K353 is the major acetylation site affecting HSPA5 ubiquitination.

Direct competition between lysine acetylation and ubiquitination has been proposed as a major regulatory mechanism to prevent protein ubiquitination and degradation [32] and has been suggested to serve a protective role for lysine acetylation by preventing further modification of adjacent lysine residues. p300 is known to acetylate Runx3 and prevent its degradation by Smurf E3s [33], and E1A is involved in p300-mediated regulation of histone activity in cell transformation [34]. Thus, we hypothesized that p300 also plays a role in E1A-mediated HSPA5 inhibition. As shown in Fig. 4D , p300 associated with HSPA5 and enriched acetylated HSPA5 in 231/V cells. However, E1A prevented p300 binding to HSPA5, leading to a reduction in acetylated HSPA5 (Fig. 4D ). Next, we examined whether the association of E1A and p300 was required for HSPA5 acetylation regulation. HeLa cells were transfected with HSPA5 and HA-tagged p300 in E1A- or E1A mutant-expressing cells, followed by IP and Western blot analysis. p300 associated with HSPA5 in E1A mutant-expressing cells but not in wild-type E1A-expressing cells (Fig. 4E ). These findings suggest that p300 binds to and acetylates HSPA5 and that E1A expression abolishes the p300 binding to HSPA5 required to promote the ubiquitination and subsequent degradation of HSPA5.

### HSPA5 acetylation is mediated by p300 at K353

**Figure 5:**
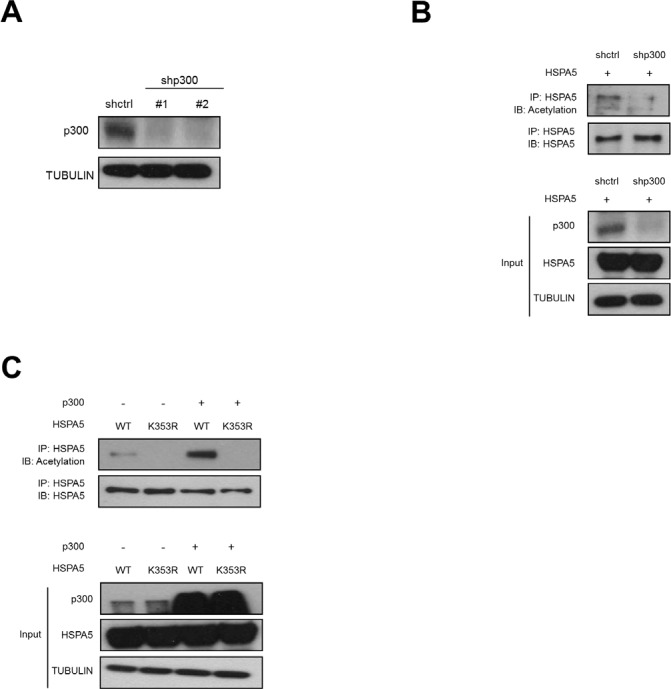
p300-dependent acetylation of HSPA5 at K353 (A) Hela cells were transfected with two p300 specific shRNAs (shp300#1, shp300#2) and control shRNA (shctrl) and analyzed the protein expression of p300 by Western blot analysis. TUBULIN was used as a loading control. (B) shctrl cells and shp300 cells were transfected with HSPA5 expression vector followed by IP and Western blot assays. (C) HeLa cells were transfected with HSPA5 wild type or HSPA5/K353R mutant construct in the presence or absence of p300 expressing vector, cell lysates were harvested for IP and Western blot assays.

To investigate whether p300 is the acetyltransferase for HSPA5, we used two different shRNAs to knockdown p300 expression (Fig. 5A ) and examined HSPA5 acetylation. As shown in Fig. 5B , depletion of p300 significantly inhibited HSPA5 acetylation. Moreover, we transfected p300 with HSPA5 wild-type and HSPA5/K353R mutant constructs and then examined the acetylation status of HSPA5. p300 expression increased HSPA5 acetylation in wild-type HSPA5 expressing clones but not in HSPA5/K353R mutant clones (Fig. 5C ). The above results demonstrate that p300 is the acetyltransferase for HSPA5 and that the K353 residue is critical for p300-mediated HSPA5 acetylation.

## DISCUSSION

**Figure 6:**
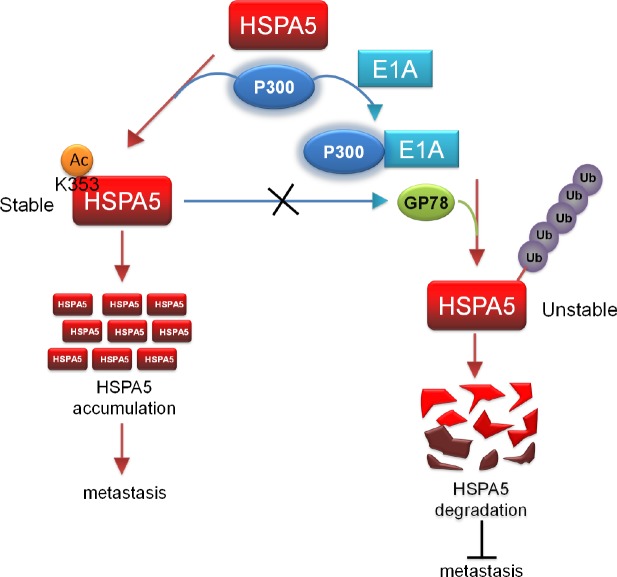
p300/E1A complex-regulated HSPA5 deacetylation is critical for subsequent HSPA5 proteolysis and inhibition of metastasis A proposed model describes the posttranslational modification of HSPA5. Acetylation of HSPA5 by p300 at K353 lysine residue is critical for HSPA5 accumulation and promotes breast cancer metastasis. p300/E1A complex deacetylates HSPA5 and induces HSPA5 ubiquitination then inhibit cancer metastasis.

HSPA5 is an ER chaperone that assists in the degradation of misfolded and accumulated proteins and controls the content and accuracy of proteins. HSPA5 overexpression is frequently observed in many types of human tumors. Our study revealed that GP78 E3 ubiquitin ligase decreased HSPA5 stability but did not affect its acetylation. When E1A was expressed in cancer cells, it associated with p300 to reduce acetylated HSPA5 levels and enhanced its binding to GP78, thereby promoting the HSPA5 ubiquitination and subsequent inhibition of metastasis (Fig. 6 ). The mechanism of action is controlled by the competitive acetylation of K353 and ubiquitination of HSPA5, revealing a new mechanism for the posttranslational regulation of HSPA5 expression.

E1A-mediated degradation of HSPA5 blocks cell transformation may through UPR-independent pathway. HSPA5 belongs to the glucose-regulated proteins (GRPs) family, which includes the stress inducible molecular chaperones GRP94, GRP170 and GRP75 [35]. With HSPA5 depletion or inactivation, UPR can be spontaneously triggered through other molecules, which illustrates that HSPA5 is not the only molecule mediating UPR. When one mediator decreases, such as HSPA5, other chaperons, such as GRP94, can maintain the UPR response [35]. HSPA5 has different effects on cell transformation and associates with aggressive growth and invasive properties in various tumor models [36-41]. HSPA5 knockdown has also decreased cancer cell invasion *in vitro* and metastasis in a mouse model [37, 38]. HSPA5 overexpression increases the activity of focal adhesion kinase (FAK) and promotes the invasion of hepatocellular carcinoma cells both *in vivo* and *in vitro* [39]. Surface HSPA5 can bind to alpha2-macroglobulin (alpha2M*) to activate PAK-2 and promote metastasis in prostate cancer [40]. HSPA5 can also bind to uPAR to activate uPA and plasminogen, facilitating cell migration and invasion [41]. The above studies provide the possibility that E1A-mediated degradation of HSPA5 blocks cell transformation through an UPR-independent pathway.

Acetylation and phosphorylation have been previously suggested to control the activity of the heat shock protein family, including HSP90 [42, 43] and HSPA5 [31]; however, competitive regulation through acetylation and ubiquitination has not been demonstrated. Three major mechanisms are involved in the control of protein stability following lysine acetylation. First, competition-based protein stabilization with direct competition by ubiquitin and acetyl groups on the same lysine residues regulates protein stability. Second, acetylation-dependent protein stabilization, where lysine acetylation in a protein creates a high-affinity-binding site for other proteins, such as E3 ubiquitin ligase, promotes ubiquitination. In contrast, a given acetylated lysine may attract a partner to mask other lysines and protect them from E3 ubiquitin ligase, preventing degradation. Third, lysine acetylation may lead to complex dissociation and would thereby render their components accessible to the action of protein degradation machinery and promote complex dissociation [32]. Our observations suggest that HSPA5 acetylation at K353 prevents ubiquitination and degradation of HSPA5. This finding supports direct competition between lysine acetylation and ubiquitination as a major mechanism regulating HSPA5 degradation. Indeed, a protective role for lysine acetylation by preventing further modifications has been suggested for crucial regulatory factors, such as p53, p73, NF-E4, and Runx3. The same residues on p53 are the targets of protein acetylation and ubiquitination; however, E1A expression stabilizes p53, disrupting its ubiquitination through the association of p300 and E1A [44]. Furthermore, the crosstalk between protein acetylation and ubiquitination also has the potential to affect transcription and intracellular trafficking. For instance, while p53 ubiquitination is tightly controlled by its acetylation, the regulatory mechanism is related not only to p53 stability but also to its subcellular localization (nuclear export) [45]. Recently, beyond the ER, mitochondrial, nuclear, cytoplasmic and surface forms of HSPA5 have been linked to cellular homeostasis and therapeutic resistance [46]. The crosstalk between HSPA5 acetylation and ubiquitination regulating protein stability, which we identified in this study, may further investigates a role in HSPA5 cellular localization.

E3 ubiquitin ligase is the critical molecule in the ubiquitin proteasome system that is responsible for substrate specificity. Previous studies have indicated that GP78 recognizes its substrate CYP3A4 via protein phosphorylation [47]. However, we identified a different mechanism for GP78 substrate recognition involving competitive acetylation and ubiquitination that is critical for HSPA5 stability. Based on our study, E1A-mediated GP78 E3 ubiquitin ligase activity acts as a tumor suppressor through HSPA5 regulation. Interestingly, our *in vivo* results suggest that GP78 negatively regulates tumor metastasis and tumor growth in response to E1A through the HSPA5 ubiquitination.

Recently, studies have confirmed that phosphoinositide 3-kinase (PI3K) is a key player in breast cancer pathogenesis and that the inhibition of its downstream AKT activity has the potential to decrease breast cancer progression [48]. Interestingly, knockdown of HSPA5 blocks downstream PI3K/AKT signaling and has been reported to decrease prostate and leukemia tumorigenesis [13, 37]. In our study, we demonstrated that E1A significantly decreased HSPA5 expression in three breast cancer cell lines, MDA-MB-231, HBL100 and HS578T cells. The identified mechanism suggests that targeting HSPA5 for degradation may be a potential therapeutic option for treating breast cancer.

## MATERIALS AND METHODS

### Reagents and antibodies

Polyethyleneimine (PEI), cycloheximide (CHX) and MG132 were purchased from Sigma-Aldrich. HDAC inhibitor (LBH589) was purchased from BioVision (Milpitas). Matrigel was purchased from BD Biosciences (Franklin Lakes). Protease inhibitor cocktail and FuGENE 6 were purchased from Roche (Basel). All cell culture-related reagents were purchased from Invitrogen (Carlsbad). The following antibodies were used: E1A (BD Biosciences), GP78 (Santa Cruz Biotechnology) for western blot analysis, GP78 (GeneTex) for immunohistochemical staining, HSPA5 (Santa Cruz Biotechnology) for Western blot analysis, HSPA5 (Abcam) for immunohistochemical staining, p300 (Santa Cruz Biotechnology), Acetylated-Lysine (Cell Signaling), UB (Santa Cruz Biotechnology), HA (Roche), MYC (Roche), FLAG (Sigma-Aldrich) and Tubulin (Sigma-Aldrich). All secondary antibodies were purchased from Jackson Immuno Research (West Grove).

### Cell culture and transfection

Human breast cancer cell lines (MDA-MB-231, HS578T and HBL100) were purchased from the American Type Culture Collection (ATCC) and grown in Dulbecco's Modified Eagle's Medium (DMEM)/F12 supplemented with 10% FBS. The human breast cancer cell line MDA-MB-231 and its E1A/vector stable transfectants have been described previously [20].

### Construct of expression vectors and plasmids

Full-length human HSPA5 (NM_005347) was amplified by PCR using cDNA of HeLa cells and cloned into the *BamH*I and *Xho*I site of pcDNA6/MYC-His (Invitrogen). The N-terminal and C-terminal deletion constructs of HSPA5 were further amplified from full-length human HSPA5 and cloned as described above. Full-length human GP78 (NM_001144) was amplified by PCR using cDNA of HeLa cells and cloned into the *BamH*I and *Xba*I site of HA tagged pcDNA3 (Invitrogen). Full-length human Ubiqutin (UB) was amplified by PCR using pRK5-HA-Ubiquitin (Addgene plasmid 17608) as template and cloned into the *EcoR*V and *Xba*I site of pcDNA6/MYC-His (Invitrogen). The HSPA5 mutation (K352R, K353R and K2R), and E1A mutation (E1A/Mut) were mutated using a QuickChange II XL Site-Directed Mutagenesis Kit (Stratagene). All primers sequences of cDNA and mutation constructs are shown in Supplementary Table S1. The lowercase was representative to the additional sequence. Restriction enzyme site and mutation site sequences were underlined. All constructs were confirmed by DNA sequencing. The lentiviral HSPA5 shRNA clones TRCN0000218611 (#1) and TRCN0000231123 (#2), the p300 shRNA clones TRCN0000009882 (#1) and TRCN0000078630 (#2) the pLKO.1-shLuc vector TRCN0000072244 that was shRNA against luciferase act as a control, the pMD2.G plasmid and pCMVdeltaR8.91 plasmid were purchased from the National RNAi Core Facility at Academia Sinica, Taipei, Taiwan. The shGP78, shCHIP, shCUL5 and shPARKIN plasmids were provided by Prof. Michael Hsiao (Genomics Research Center, Academia Sinica, Taipei, Taiwan) as kindly gifts. Plasmid pUK21-CMV-E1A was described previously [49]. The E1A mutant carries point mutation (F66A and D68A) in conserved region 1 (CR1), the critical motif be recognize by p300 transcriptional adaptor motif (TRAM), to prevent the interaction between p300 and E1A [50] and pCI-Neo-GP78 R2M (Addgene plasmid 13304), pCMVb p300 HA (Addgene plasmid 10718) and pRK5-HA-Ubiquitin (Addgene plasmid 17608) were obtained from Addgene (http://www.addgene.org/).

### RNA isolation, reverse transcription and real-time PCR quantification

Total RNA was isolated using Trizol reagent (Invitrogen) and reverse transcribed into cDNA using M-MLV reverse transcriptase (Invitrogen) according to the manufacturer's instructions. Real-time PCR was performed using the Roche LightCycler 480 (Roche). For mRNA detection, PCR reactions contained 0.5 μM of each forward and reverse primer, 1 μM Universal ProbeLibrary Probe (Roche), 1 × LightCycler TaqMan Master mix, and 2 μl of cDNA. Amplification curves were generated with an initial denaturing step at 95°C for 10 min, followed by 50 cycles of 95°C for 5 s, 60°C for 10 s, and 72°C for 1 s. All primers sequences of mRNA and probe are shown in Supplementary Table S1. The *GAPDH* was used as the reference gene. The relative levels of gene expression were represented as ΔCt = Ct gene – Ct reference, and the fold change of gene expression was calculated by the 2^−ΔΔCt^ Method.

### Protein stability assay and Western blot analysis

For protein stability assays, cells were incubated with cycloheximide (CHX, Sigma-Aldrich) to inhibit further protein synthesis and incubated with MG132 (Sigma-Aldrich) to inhibit 26S proteasome for the indicated times, cells were immediately harvested. Proteins in the total cell lysates were separated on SDS-PAGE and electrotransferred to a polyvinylidene difluoride membrane (Millipore). After blocking, blots were incubated with specific primary antibodies, and after washing and incubating with secondary antibodies, immunoreactive proteins were visualized using an enhanced chemiluminescence detection system (PerkinElmer, Waltham).

### Immunoprecipitation

Cells were lysed by brief sonication in co-immunoprecipitation buffer (20 mM Tris-HCl [pH 7.4], 150 mM NaCl, 1 mM CaCl_2_, 2 mM MnCl_2_, 1.2% Triton X-100) supplemented with protease inhibitors cocktails (Roche). Lysates were centrifuged for 30 min at 14,000 × *g* and the resulting supernatant was precleared by incubation with Protein A (for rabbit antibody) or G (for mouse antibody) for 1 h at 4°C. The precleared supernatant was subjected to overnight immunoprecipitation using the indicated antibodies or control IgG antibodies at 4°C. The next day, protein complexes were collected by incubation with Protein A (for rabbit antibody) or G (for mouse antibody) for 1 h at 4°C. The collected protein complexes were washed five times with co-immunoprecipitation buffer and eluted by boiling in protein sample buffer under reducing conditions, after which proteins were resolved by SDS-PAGE and analyzed by Western blotting.

### Lentivirus infection

Recombinant lentiviruses were produced by co-transfecting a mixture of the indicated expression plasmid, the envelope plasmid (pMD2.G) and the packaging plasmid (pCMVdeltaR8.91) into HEK293T cells using PEI (Sigma-Aldrich) according to the manufacturer's instructions. The viruses were harvested from the culture medium on day 2 after transfection and filtered with a 0.45 μm filter. Cultured cells were incubated with lentivirus containing 8 μg/ml polybrene for 24 h, replaced fresh medium and incubated for another 48 h. For stable cell lines, cells were selected by puromycin.

### Transwell migration and invasion assays

For transwell migration assays, 1 × 10^5^ or 5 × 10^4^ cells were plated in the top chamber onto the non-coated membrane (24-well insert; pore size, 8 μm; Corning Costar). For invasion assay, 1 × 10^5^ or 5 × 10^4^ cells were plated in the top chamber onto the matrigel-coated membrane and the detailed procedure have been described previously [26]. The numbers of cells that migrated and invaded were normalized to the proliferation by MTT assay for each cell line.

### Cell tracing using a time-lapse microscope imaging system

5 × 10^4^ cells were plated in the 6 cm dish the day prior to performing the cell tracing assay. The cells were then placed into the incubation container at 37°C in a humidified atmosphere at 5% CO_2_ which is placed on the stage of a light microscope (Zeiss). Seventy-five pictures of living cells were taken every 15 min at 50 × magnification. To analyze these data, Image J (NIH), was used to count and track cells according to manufacturer instructions. We took at least 20 single cells to calculate the whole path distance to represent the migration ability of these cells.

### Animal studies

All animal work was performed in accordance with protocols approved by the Institutional Animal Care and Use Committee of China Medical University. Female severe combined immunodeficient (SCID) mice (supplied by LASCO, Taiwan), age matched and 4 to 6 weeks old, were used in assays for lung colonization metastasis in an experimental metastasis model. For experimental metastasis assays, 3 × 10^5^ viable cells were resuspended in 0.1 ml of PBS and introduced into the circulation via tail-vein injection. Lung metastasis was quantified 8 weeks after injection. The luciferase-based, noninvasive bioluminescent imaging and analysis were performed by the Xenogen IVIS-200 system (Xenogen, Alameda). The remaining mice from each group were monitored for survival studies.

### Statistical analysis

All analyses were performed using SPSS software (version 13.0 for Windows; SPSS Inc.). All data are presented as the mean ± standard error (s.e.m.) from at least three independent experiments. The Student's *t*-test was used to compare data between two groups. Statistical analyses of inverse correlation between HSPA5 and GP78 were performed using Spearman's nonparametric correlation test. Survival curves were obtained using the Kaplan-Meier method, and the log-rank test was used to test the difference in survival curves. *p* values of less than 0.05 were considered to be statistically significant.

## SUPPLEMENTARY MATERIAL TABLE


