# Supplementary material for: De-acetylation and degradation of HSPA5 is critical for E1A metastasis suppression in breast cancer cells
Source: Oncotarget. 2014 Sep 25;5(21):10558–70. doi: 10.18632/oncotarget.2510 (PMC4279393; doi:10.18632/oncotarget.2510)
Supplement: Supplementary file 1 [file oncotarget-05-10558-s001.pdf]

De-acetylation and degradation of HSPA5 is critical for E1A metastasis suppression in breast cancer cells

Supplementary Material

Supplementary Table S1: Sequences and information of PCR primers for mRNA and cloning

**mRNA PCR primers**
HSPA5
5'-AGCCTGGCGACAAGAGTG-3' (forward)
5'-AATCCAATACTGCCCAAGGA-3' (reverse)
Universal ProbeLibrary Probe #39
GP78
5'-CTACACCCACGGAATGCAC-3' (forward)
5'-GCAGTCCTCACTGTCACAAGA-3' (reverse)
Universal ProbeLibrary Probe #46
CHIP
5'-AGGCCAAGCACGACAAGTA-3' (forward)
5'-CAGGTAGTCGGGGATGTCTC-3' (reverse)
Universal ProbeLibrary Probe #35
CUL5
5'-AGAATGTAACTCCGTTGAAGCAC-3' (forward)
5'-ATGCCTTGGCACTCAGCTA-3' (reverse)
Universal ProbeLibrary Probe #37
PARKIN
5'-GGAGCTGAGGAATGACTGGA-3' (forward)
5'-ACAATGTGAACAATGCTCTGCT-3' (reverse)
Universal ProbeLibrary Probe #85
GAPDH
5'-AGCCACATCGCTCAGACAC-3' (forward)
5'-GCCCAATACGACCAAATCC-3' (reverse)
Universal ProbeLibrary Probe #60
**Cloning primer**
HSPA5
5'-ggggatccATGAAGCTCTCCCTGGTGG-3' (forward)
5'-ggctcgagCAACTCATCTTTTTCTGCTG-3' (reverse)
GP78

5’-ggggatccATGCCGCTGCTCTTC-3’ (forward)
5’-ggtctagaCTAGGAGGTCTGCTGCTTCTG-3’ (reverse)
Ub
5’-ATGGATTACAAGGATGACGACGATAAGATGCAGATTTTCGTGAA-3’ (forward)
5’-ggtctagaTTAACCACCTCTCAGAC-3’ (reverse)
HSPA5 (K352R)
5’- CCAGAAAGTGTTGGAAGATTCTGATTTGAGGAAGTCTGAT-3’ (forward)
5’-ATCAGACTTCCTCAAATCAGAATCTTCCAACACTTTCTGG-3’ (reverse)
HSPA5 (K353R)
5’-CGTCCAGAAAGTGTTGGAAGATTCTGATTTGAAGAGGTCTGATATTG-3’ (forward)
5’-CAATATCAGACCTCTTCAAATCAGAATCTTCCAACACTTTCTGGACG-3’ (reverse)
HSPA5 (K2R)
5’-CCAGAAAGTGTTGGAAGATTCTGATTTGAGGAGGTCTGAT -3’ (forward)
5’-ATCAGACCTCCTCAAATCAGAATCTTCCAACACTTTCTGG-3’ (reverse)
E1A (F66A, D68A)
5’-GGTTTCGCAGATTGCTCCCGCCTCTGTAATGTTGGC-3’ (forward)
5’-GCCAACATTACAGAGGCGGGAGCAATCTGCGAAACC-3’ (reverse)
